# The Daily Experiences of Hispanic and Latinx Dementia Caregivers Study: Protocol for a Fully Remote Daily Diary Observational Cohort Study

**DOI:** 10.2196/55216

**Published:** 2024-06-13

**Authors:** Sofía Mildrum Chana, Lorelí Álvarez, Abigail Poe, Natashia Bibriescas, Danny Hai Wang, Stephanie DiFiglia, Andrés Azuero, Michael Crowe, Frank Puga

**Affiliations:** 1 Department of Psychology University of Alabama at Birmingham Birmingham, AL United States; 2 Department of Acute, Chronic and Continuing Care School of Nursing University of Alabama at Birmingham Birmingham, AL United States; 3 Department of Educational Psychology University of Texas at Austin Austin, TX United States; 4 Department of Biobehavioral Health, College of Health and Human Development The Pennsylvania State University University Park, PA United States; 5 MJHS Institute for Innovation in Palliative Care New York, NY United States; 6 Department of Nursing Family, Community & Health Systems School of Nursing University of Alabama at Birmingham Birmingham, AL United States

**Keywords:** dementia, caregivers, Hispanic, Latinx, mental health, daily diary, longitudinal, protocol, observational cohort study, cohort, Alzheimer’s disease, Alzheimer, stress, burden, mental health, loneliness, well-being

## Abstract

**Background:**

The Hispanic and Latinx community is disproportionately affected by Alzheimer disease and related dementias (ADRDs). In the United States, approximately 8.5 million caregivers of individuals with ADRDs identify as Hispanic and Latinx people, and caregiving-related stress and burden place caregivers at elevated risk for poor mental health outcomes, as well as loneliness and social isolation. To date, there is limited knowledge about the daily stress experiences of Hispanic and Latinx caregivers. Given this knowledge gap, it is critical to examine how personal, cultural, and contextual factors influence daily stress, mental health, and resilience over time among Hispanic and Latinx ADRD caregivers.

**Objective:**

The goal of this protocol report is to present the rationale, methodology, planned analytical strategy, progress completed to date, and implications of future findings for “Nuestros Días” (Spanish for “our days”), a fully remote daily diary (DD), observational cohort study examining the day-to-day experiences of Hispanic and Latinx ADRD caregivers.

**Methods:**

The study will recruit a cohort of up to 500 Hispanic and Latinx caregivers of individuals living with ADRD. Participants will complete measures assessing contextual, individual-level, and cultural factors at 3 intervals (enrollment, 6 months, and 12 months). Each of the timepoints will be followed by 21 days of DD surveys to report on daily stress, stress moderators, and mental health variables.

**Results:**

Data collection began in March 2023 and is projected to end in December 2026. As of March 2024, we have enrolled 60 caregivers in the Nuestros Días study, 78.9% (n=15) of whom are Spanish speakers. The current completion rate for DD surveys is 79.4%, averaging approximately 18 surveys out of 21 completed. We expect to enroll 10 to 15 participants per month moving forward to achieve our enrollment goal.

**Conclusions:**

Results from this study will identify which Hispanic and Latinx ADRD caregivers, and under what circumstances, appear to be at the greatest risk of experiencing poor mental health outcomes over time. This study represents a critical step forward in providing key guidance to develop effective, culturally sensitive interventions to support the health and well-being of Hispanic and Latinx ADRD caregivers, a historically underrepresented and underserved population in aging and caregiving research.

**International Registered Report Identifier (IRRID):**

DERR1-10.2196/55216

## Introduction

### Background

The prevalence of Alzheimer disease and related dementias (ADRDs) among the Hispanic and Latinx populations is 1.5 times higher than among non-Hispanic White populations [1]. Hispanic and Latinx adults exhibit an earlier onset of ADRDs and more severe symptoms at the time of diagnosis than other ethnic and racial groups [2,3]. Moreover, the presence of comorbidities, such as diabetes, heart disease, and hypertension, among Hispanic and Latinx individuals with ADRDs introduces further health complexities, exacerbating ADRD-related symptoms and accelerating functional decline [4]. Despite facing worse health outcomes and increased disability, nursing home admissions are lower among older Hispanic and Latinx individuals, suggesting increased reliance on informal caregiving [5].

Notably, Hispanic and Latinx ADRD caregivers exhibit poorer mental health outcomes compared with non-Hispanic White ADRD caregivers [6-9]. However, a limited understanding of the daily stressors and dynamic predictors of changes in mental health over time exists. Prior investigations into the mental health of Hispanic and Latinx ADRD caregivers have reported inconsistent results, likely due to varying quality of evidence across studies, which highlights the need for more in-depth research [10]. This inconsistency may arise from dynamic interactions between contextual, individual-level, and unique cultural factors contributing to within-person variability.

Conventional between-group designs and cross-cultural comparisons may not fully capture such within-group variability, thus potentially masking important factors driving mental health outcomes over time. Previous studies on caregiver mental health have found daily variations in mental health [11]. The emerging evidence of daily mental health experiences exemplifies the necessity to examine day-to-day variations in these experiences—especially among populations that may be disproportionately impacted by ADRDs and caregiving-related stress.

To address this gap, the Nuestros Días (Spanish for “our days”) study implements a daily diary (DD) approach, offering a novel and comprehensive method to understand the day-to-day stress and mental health experiences of Hispanic and Latinx ADRD caregivers. Daily diaries provide granular data that can determine intricate patterns and nuances that might be otherwise missed in broader survey methods [12]. Examining daily experiences can reveal how specific events or triggers may lead to heightened stress or emotional arousal, allowing for a more holistic understanding of the factors influencing an individual’s well-being. As such, when studying the mental health of Hispanic and Latinx ADRD caregivers, DD studies can provide invaluable insights and further directions for ecologically relevant and culturally tailored interventions to support the well-being of Hispanic and Latinx ADRD caregivers.

### Study Aims

#### Overview

The following report presents the design for the Nuestros Días study, a comprehensive and innovative investigation involving Hispanic and Latinx ADRD caregivers, who will be asked to complete measures assessing contextual, individual-level, and cultural factors at 3 intervals within a year (enrollment, 6 months, and 12 months). Subsequently, participants will engage in 21 days of diary surveys measuring daily caregiving experiences and mental health outcomes. The DD methodology encompasses various assessment techniques enabling researchers to investigate individuals’ experiences, behaviors, and situations in their natural environments, nearly in real time, and through repeated measurements spanning from a few days to months. Daily diaries have been previously used to quantitatively examine the day-to-day experiences of dementia caregivers including daily stress and well-being [13-15]. Overall, this research design enables the exploration of intraindividual variability (eg, fluctuations in daily experiences) and interindividual differences (eg, variations within distinct groups such as those with high levels of familism or religiosity vs those with low levels) and potential relationships among them [16]. Moreover, this design facilitates the identification of distinct groups within a population that aggregate-level estimates may not accurately capture [16,17]. The 3 study aims are as follows.

#### Examine Factors Influencing Daily Mental Health Experiences

The objective of this aim is to identify daily factors that increase (ie, risk factors) or decrease (ie, protective factors) the daily odds of experiencing moderate or severe depression and anxiety symptoms among Hispanic and Latinx ADRD caregivers.

#### Characterize Mental Health Developmental Trajectories

The objective of this aim is to investigate the developmental course of depression- and anxiety-related symptoms among Hispanic and Latinx ADRD caregivers over 12 months.

#### Identify Predictive Factors for Distal Health Outcomes

The objective of this aim is to examine the association between developmental trajectories of depression- and anxiety-related symptoms with levels of resilience and with distal health outcomes (eg, overall physical, mental, and social health) among Hispanic and Latinx ADRD caregivers.

## Methods

### Overview of the Study Design

This multi-time scale study will test the hypothesis that contextual, individual-level, and cultural factors influence daily and long-term mental health patterns among Hispanic and Latinx ADRD caregivers. Participants (N=500) will be asked to complete surveys measuring contextual, individual-level, and cultural factors at 3 time points (ie, enrollment, 6 months, and 12 months). Immediately after completing each survey, participants will complete 21 days of DD surveys evaluating caregivers’ day-to-day caregiving experiences and mental health (for a total of 63 diary surveys across the 3 sampling periods).

### Ethical Considerations

The study procedures were approved by the University of Alabama at Birmingham institutional review board (IRB-300008710). The study was deemed to be minimal risk, and a waiver of written consent was approved. Participants will be provided a study information sheet with details about the study’s purpose, procedures, risks, and contact information. Participants are informed that all data will be kept deidentified and that no questions regarding immigration status will be asked.

### Participant Eligibility Criteria

The target population for the study is English- and Spanish-speaking Hispanic and Latinx ADRD caregivers across the United States. The sample eligibility criteria are guided by AARP’s definition of family caregivers [18] and the sampling frame for family caregivers used by the National Institute on Aging–funded REACH trials [19].

Inclusion criteria are as follows: (1) the caregiver is 18 years or older; (2) the caregiver identifies as Hispanic or Latinx descent; (3) the caregiver provides informal care to a spouse/common-law partner, parent, or grandparent (or in-law; 60 years or older), living with dementia; (4) the caregiver assists with at least 2 instrumental activities of daily living or 1 activity of daily living; and (5) the caregiver lives with or shares cooking facilities with the care recipient.

The target sample includes any type of dementia (ie, Alzheimer and frontotemporal dementia) reported by the caregiver, and this will be accounted for in the analytic models. The AD8 questionnaire is being used to determine dementia eligibility status [20], as this is a valid and reliable measure with excellent discriminant validity to distinguish between normal cognition and mild cognitive impairment or dementia. A cutoff score of 2 or greater (ie, cognitive impairment is likely to be present) on the AD8 will be used to determine eligibility. Participants will have the option of completing the study in the language of their choice (ie, either English or Spanish).

Exclusion criteria are as follows: (1) admission to a nursing home or institutional care for the care recipient planned within the next 6 months, (2) the caregiver is terminally ill, and (3) the caregiver does not have reliable internet access.

### Participant Recruitment Strategies

Several strategies are in place to facilitate participant recruitment (Textbox 1). All of the recruitment materials have been developed in English and Spanish. As part of our analytical strategy, we will examine the effectiveness of each recruitment strategy including participant characteristics, reach, number of participants enrolled via each method, and costs associated with each strategy. We will also examine demographic differences that arise from the different recruitment methods used to identify possible selection biases.

**Overview of strategies to facilitate recruitment for the Nuestros Días (“our days”): the daily experiences of Hispanic and Latinx dementia caregivers study.**

**Print recruitment**

Newsletter advertisements tailored for senior centers and newspapers serving the Hispanic and Latinx communities. Advertisements include basic information about the study and contact details.

**Radio-based recruitment**

Advertisements on local Spanish-speaking radio stations in select cities. Advertisements include basic information about the study, as well as contact details.

**Digital-based recruitment**

Social media campaign with a marketing agency to program study advertisements to appear in the feed of social media sites including Facebook and Instagram. Once individuals click on the “Sign up” button in these ads, they are directed to an initial screening survey that includes questions to verify if the user meets the study eligibility criteria. In addition, we have developed a website for this study with support from the University of Alabama School of Nursing’s strategic marketing and communications team.

**Community outreach recruitment**

Partnership development with local organizations nationwide serving the Hispanic and Latinx community, as well as people living with dementia and their caregivers (eg, Alzheimer Association local chapters and the Area Agencies on Aging).

**Registry-based recruitment**

Registration of this study on sites that share information about ongoing research studies with potentially eligible participants (eg, Alzheimer Association’s registry, Family Caregiver Alliance’s registry).

### Remote Data Collection Procedures

#### Eligibility Screening and Enrollment

The eligibility screening process is completed via web and involves a 3-step approach and requires administrative confirmation at each step. All recruitment materials include a link to complete an initial eligibility screening questionnaire. This questionnaire includes prompts to assess whether the potential participants meet the study eligibility criteria specified above. In addition, participants’ IP addresses are recorded and screened by study personnel to prevent fraudulent responses. This screening process is supported by tools such as Scamalytics [21] or WhatIsMyIPAddress.com [22].

Study personnel then verify participants’ responses and those eligible to take part in the study receive the study information sheet with details regarding study participation. This study has a waiver of consent due to its low-risk nature, and all participants are asked to acknowledge they have reviewed the information sheet.

Afterward, participants are sent a link to complete a verification questionnaire. This questionnaire includes similar prompts as the initial eligibility screening questionnaire, and serves as an information verification step, as responses from both questionnaires are cross-referenced by study personnel. This process aims to minimize the risk of enrolling fraudulent participants, as well as potential software bots. Participants deemed eligible after this verification procedure are then called by a member of the study team to confirm their enrollment in the study as part of the third and final step. During this call, participant contact information and eligibility are verbally verified one last time. In addition, the start dates for the surveys are confirmed by the study team members during this call. This step also helps ensure that only real participants who meet the eligibility criteria are enrolled in the study.

#### Data Collection

All data are collected using REDCap (Research Electronic Data Capture; Vanderbilt University), and accessed via the REDCap Consortium. REDCap is a web-based data collection platform aimed to securely support a variety of data management tasks including data tracking, manipulation, export, and others [23,24]. Participants are sent links to complete surveys to the email address provided during enrollment. All surveys are sent at 7 PM in the participant’s respective time zone. First, they are prompted to complete a 40-60 minute baseline questionnaire (see Table 1 for measures).

**Table 1:** Brief descriptions of measures organized by construct for the traditional, longer-recall surveys that participants complete at baseline, 6 months, and 12 months in the Nuestros Días (“our days”): the daily experiences of Hispanic and Latinx dementia caregivers study.

Construct	Measure	Description
Demographics^a^	N/A^b^	Basic demographic information, including age, gender identity, sex assigned at birth, native language, country of origin, race/ethnicity, sexual orientation, marital status, education, employment, income, medical insurance, time spent as a caregiver, and relationship to the care recipient, as well as the following questions about the care recipient: age, gender identity, race/ethnicity, education, insurance, type of dementia diagnosis, and other chronic conditions.
Caregiving-related stress	BSD^c^ [25,26]	Twenty items reflecting different domains of BSD (restlessness, mood, memory, care resistance, hallucinations and delusions, verbal and physical aggression, disinhibition, repetition, safety) with stress appraisals reported on a 5-point Likert scale. Items were adapted from several established instruments because they represent the diversity of types of symptoms rather than overarching conceptual constructs.
Caregiver burden	Zarit burden inventory-short form [27]	Twelve items on a 5-point Likert scale measuring dimensions of personal strain and role strain related to caregiving (α=0.88).
Noncaregiving related stress	Perceived stress scale [28]	Ten items on a 5-point Likert scale measuring perceptions of unpredictability, lack of control, and overload in one’s life (α=0.78).
Family stress	Family caregiver conflict scale [29]	Eight items on a 4-point Likert scale measuring caregiving-related conflicts between caregivers and other family members (α=0.93).
Financial stress	Financial subscale of the caregiver reaction assessment [30]	Three items on a 6-point Likert scale measuring caregiving-related financial strain (α=0.83).
Caregiver demands	N/A	Single item asking how many hours of care per week they provide to the PLWD^d^.
Relationship quality^a^	Relationship closeness scale [31]	Six items on a 4-point Likert scale measuring relationship quality between caregiver and care recipient (α=0.90).
Coping strategies^a^	Brief-COPE Inventory^e^ [32]	Adapted scale with 14 items on a 4-point Likert scale measuring situational coping including strategies of avoidance, problem-solving, and seeking informational support at the personal level (α=0.83-0.91).
Social support	PROMIS^f^ social health SF [33-35]	Measures perceptions of emotional (4 items), informational (4 items), and instrumental support (4 items) on a 5-point Likert scale.
Social isolation	PROMIS SF^g^ social isolation scale [33,34]	Four items on a 5-point Likert scale measuring perceptions of social isolation.
Emotion regulation^a^	Emotion regulation questionnaire [36]	Ten items measuring cognitive reappraisal (6-items, α=0.83) and expressive suppression (4- items, α=0.79) using a 7-point Likert scale.
Alcohol use^a^	AUDIT^h^ [37]	Ten items measuring alcohol use (α=0.83).
Cultural values^a^	Mexican American cultural values scale [38]	Adapted scale includes 36 items on a 5-point Likert scale measuring cultural values including fatalism, familism support, familism obligation, familism reference, religion, and traditional gender roles.
Acculturation^a^	Brief acculturation scale for the Hispanic population [39]	Four items on a 6-point Likert scale measuring acculturation in language use, media, and ethnic social relations (α=0.94) [40].
Discrimination^a^	Everyday discrimination scale [41]	Nine items on a 6-point Likert scale measuring experiences of racism and discrimination (α=0.88).
Overall health	PROMIS global health scale [42]	Ten items on a 5-point Likert scale measuring overall physical and mental health.
Depressive symptoms	Patient health questionnaire [43]	Nine item–questionnaire on a 4-point Likert scale measuring severity of depression symptoms and suicidal ideation (α=0.86-0.89).
Anxiety symptoms	General anxiety disorder questionnaire [44]	Seven items on a 4-point Likert scale measuring the severity of anxiety symptoms (α=0.92).
Resilience	Resilience scale for adults [45]	Adapted scale includes 14 items on a 7-point Likert scale measuring resilience with 3 subscales (perception of self, planned future, and structured style).
Decision-making involvement	Decision-making involvement scale for individuals with dementia and family caregivers [46]	Ten items measuring the degree of a caregiver’s involvement in the everyday decisions of a person living with dementia (α=0.85).
Social media use habits	N/A	Six items assessing social media platforms of choice, duration of use, frequency of checking, and preferred device to engage with social media over the past 6 months. In addition, passive and active social media use are measured with questions based on SMAQ^i^; (*α for* active=0.845 and *α* for passive=0.853) [47].
Social media automaticity	Self-report behavioral automaticity index [48,49]	Four items measuring daily habitual behavior patterns regarding social media use (α=0.92).
Social media self-regulation	Bergen social media addiction scale, [50] the social media self-control failure scale, [51] and the generalized problematic internet use scale [52]	Three items from 3 different instruments assessing self-regulation abilities when using social media.

^a^Only administered in the baseline questionnaire.

^b^N/A: not applicable.

^c^BSD: behavioral symptoms of dementia.

^d^PLWD: people living with dementia.

^e^COPE: coping orientation to problems experienced.

^f^PROMIS: patient-reported outcomes measurement information system.

^g^SF: short form.

^h^AUDIT: alcohol use disorders identification test.

^i^SMAQ: social media activity questionnaire.

The initial DD survey period begins 1 day after completing the baseline questionnaire. Participants receive an email reminder once a day for 21 consecutive days to complete the diary surveys, and each email contains the REDCap link to complete the surveys. The diary surveys are expected to take 10-15 minutes to complete (see Table 2 for measures). The collection period for the diary surveys is set between 7 PM and 11 PM (in the participants’ respective time zones) to reduce recall bias. Participants receive 3 hourly reminders via email during the data collection window to encourage completion of DD surveys. If participants do not complete DD surveys 3 days in a row, a study personnel member reaches out to the participant via phone call or email to assess continued interest in study participation, as well as any study engagement barriers and reprogram DD surveys as appropriate.

**Table 2:** Brief descriptions of measures for the daily diary surveys organized by construct for the Nuestros Días (“our days”): the daily experiences of Hispanic and Latinx dementia caregivers study.

Construct	Measure	Description
Caregiving-related stress	BSD^a^ [25,26]	20 items reflecting different domains of BSD (restlessness, mood, memory, care resistance, hallucinations and delusions, verbal and physical aggression, disinhibition, repetition, safety) and the perceived stressfulness of each symptom reported on a 5-point Likert scale. Items were adapted from several established instruments because they represent the diversity of types of symptoms rather than overarching conceptual constructs.
Noncaregiving related stress	Daily stress inventory [53]	Adapted scale includes 7 items measuring daily stressors.
Coping strategies	Brief-COPE Inventory^b^ [32]	Fourteen items that measure self-distraction, active coping, denial, substance use, use of emotional support, use of instrumental support, behavioral disengagement, venting, positive reframing, planning, humor, acceptance, religion, and self-blame (α=0.83-0.91).
Social support	Social support scale [54]	Adapted scale includes 11 items measuring daily social support and resources (emotional support, instrumental support, and informational support).
Social isolation	PROMIS social isolation scale SF^c^ [33,34]	Four items measuring perceptions of social isolation.
Discrimination	Everyday discrimination scale [41]	Nine-item scale measuring experiences of racism and discrimination (α=0.88).
Depression symptoms	PROMIS emotional distress—depression scale SF^c^ [33]	Four items measuring common symptoms associated with depression.
Anxiety symptoms	PROMIS emotional distress—anxiety scale SF^c^ [33]	Four items measuring common symptoms associated with anxiety.
Suicidality	Paykel suicide scale [55]	Adapted scale includes 5 items measuring daily suicidal ideation.
Social media use habits	N/A^d^	Six items assessing daily use of social media, including platforms used, preferred platform, time spent on social media, and passive and active social media use behaviors.
Social comparison	N/A	Two items based on previous literature [56] that assess social comparison when using social media and feelings experienced afterward.

^a^BSD: behavioral symptoms of dementia.

^b^COPE: coping orientation to problems experienced

^c^SF: short form.

^d^N/A: not applicable.

**Figure 1:**
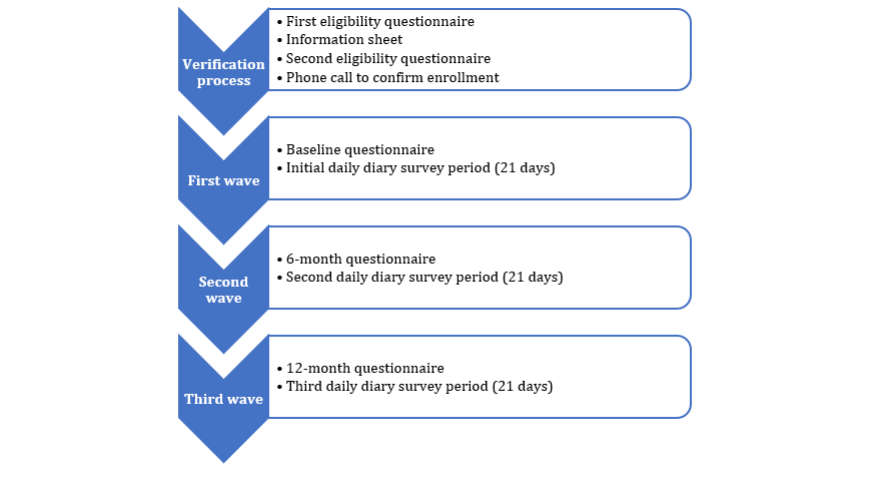
Overview of the data collection schedule for the Nuestros Días (“our days”): the daily experiences of Hispanic and Latinx dementia caregivers study. Participants first complete a screening and verification process to determine eligibility and identify potentially fraudulent responders. Then, participants completed 3 waves of data collection (baseline, 6 months, and 12 months). At each wave, participants complete traditional, longer-recall surveys measuring contextual, individual-level, and cultural factors followed by 21 days of daily diary surveys measuring day-to-day caregiving and mental health experiences.

A total of 6 months following the baseline questionnaire and initial diary study, participants repeat this process in the second wave of data collection with a 6-month follow-up questionnaire and a second set of 21 DD surveys. In the third wave of data collection, participants repeat this process 12 months after the initial wave of data collection in addition to a third set of 21 DD surveys (Figure 1).

### Data Analysis Plan

All data will be exported from REDCap and analyzed using various statistical software platforms, for example, SAS (SAS Institute Inc), SPSS (IBM Corp), R (R Core Team), and STATA. (StataCorp). First, univariate and bivariate approaches will be used to examine outstanding issues with the DD survey data including outliers and missing data. Descriptive statistics and data distributions will be evaluated to ensure values are within reasonable limits and evaluate potential issues of nonnormality and collinearity. A false discovery rate approach [57] will be used to adjust for multiple inferences when appropriate. To ensure the validity and reliability of the proposed measures in our sample, we will perform psychometric testing on all measures as part of our analytical strategy including measures that were adapted for the purposes of this study. The main analytical approaches for each of the study’s specific aims are detailed below.

For aim 1, the objective is to identify daily protective and risk factors that impact the daily odds of depression and anxiety symptoms among Hispanic and Latinx ADRD caregivers. Generalized linear mixed modeling (GLMM) accounting for the nesting of diary survey data within participants will be used to examine associations between contextual, individual-level, and cultural factors and daily mental health (ie, depression and anxiety-related symptoms).

In aim 2, the main goal is to investigate the developmental course of depression and anxiety symptoms among Hispanic and Latinx ADRD caregivers over 12 months. To achieve this, we will use group-based trajectory modeling (GBTM) [58] to identify clusters (ie, trajectory groups) of daily mental health symptom occurrence over time and determine personal and cultural factors that predict group membership. We will follow current best practices in GBTM [59] for model selection.

For aim 3, the purpose is to examine the association between developmental trajectories of depression and anxiety symptoms with levels of resilience and distal health outcomes. We will use regression modeling with the trajectories identified in aim 2 and posterior probabilities to make prognostic predictions of overall physical health and clinically significant depression and anxiety at 12 months. We will also examine levels of resilience at baseline, 6- months, and 12 months as a time-varying covariate for these relationships [58].

### Sample Size and Power Considerations

For the planned aim 1 analyses, the design proposed in this study will collect a maximum of 31,500 data points across 500 participants with 63 observations per participant (21 days x 3 sampling periods). Assuming a 0.2 uniform probability of dropout over the 12-month study period and an average of 80% diary completion, the expected datapoint collection is 22,680 datapoints (500×16.8+450×16.8+400×16.8). Further assuming, 2 equally sized groups, an intraclass correlation of 0.4 (ie, average correlation between any 2 repeated measurements on the same individual), a standardized outcome (eg, stress and depression), and an adjusted significance level of 0.025, the expected effective sample provides 80% power to detect a time-averaged between-group difference (*d*) of 0.19 [60]. Under similar assumptions for intraclass correlation and significance level, regarding within-cohort changes in standardized outcomes between sampling periods (eg, baseline vs 6 months, each with up to 21 observations per participant) the expected effective sample size provides 80% power to detect a within-cohort change (*d*) of 0.04.

For aims 2 and 3, there are no established guidelines for assessing power or determining appropriate sample sizes for GBTM as these are exploratory analyses and the number of resulting trajectories is not known a priori. In previous studies [61] evaluating depression symptoms, discrimination, and ethnic identity among Latinos, a sample of 91 participants was sufficient to detect variations in depressive symptoms. In another study [62] using GBTM, a sample of 94 caregivers of individuals with malignant brain tumors was sufficient for a 2-group trajectory model of caregiver depression and anxiety. Considering that precision is expected to increase with a greater number of data points [63], we expect the target sample size of 500 participants will yield enough precision and power for our planned analyses. Further, GBTM falls under the umbrella of growth mixture models. Previous literature on growth mixture models suggests that a sample size of 500 is sufficient for the Bayesian information criterion and sample-size adjusted Bayesian information criterion to accurately identify the correct number of latent growth trajectories [64].

## Results

We started recruitment and data collection in March 2023. Enrollment numbers following 12 months of recruitment are shown in Table 3. We have enrolled 60 family caregivers in this study, most of them are Spanish speakers (75%). Overall, 51 participants have completed the first wave of data collection and 5 are currently completing data collection, either for the baseline extended survey or DD surveys. A total of 5 participants withdrew from the study after enrollment; 3 of them did not complete the baseline survey and 1 stopped responding to the daily surveys; the research team was not able to contact these individuals via phone or email, so we do not have any further information on the reason for withdrawal; 1 participant requested to be removed for the study because of personal reasons. As shown in Table 3, the completion rate for the DD surveys is 84%, equivalent to 900 daily surveys completed for the whole sample.

**Table 3:** March 2023 through March 2024 enrollment data for the Nuestros Días (“our days”): the daily experiences of Hispanic and Latinx dementia caregivers study.

Characteristic			Participants (n=60), n (%)
**Study implementation numbers**			
	English speakers	15 (25)	
	Spanish speakers	45 (75)	
	Participants in progress	4 (7)	
	Participants completed wave 1	51 (85)	
	Participants withdrawn	5 (8)	
**Overall DD^a^ data collection progress**			
	Total participants started DDd	51 (85)	
	Total number of DDs completed	900 (84)	
	Total number of DDs expected	1071 (100)	
	Completion rate	84	

^a^DD: daily diary.

Participants who have completed the first wave of data collection responded to 18 DD surveys on average, out of the 21 DD surveys expected. Our goal is to have 70 participants by the end of April 2024. We estimate to enroll 10-15 participants per month from this date forward to successfully accrue our yearly recruitment goals.

## Discussion

### Principal Results

This study addresses a critical gap in the literature by implementing an innovative design and analytical strategy to test the hypothesis that contextual, individual-level, and cultural factors influence daily and long-term patterns of mental health outcomes among Hispanic and Latinx ADRD caregivers. With cases of ADRDs rapidly increasing among Hispanic and Latinx individuals [8,65,66], the number of Hispanic and Latinx caregivers is consequently growing. Given that Hispanic and Latinx adults develop ADRDs at a younger age and experience complex comorbid conditions, as well as more severe symptomatology than other racial or ethnic groups, Hispanic and Latinx caregivers are uniquely susceptible to increased caregiving stress that can negatively impact mental health outcomes. Thus, a better understanding of the dynamic interactions between risk and protective factors will contribute to the identification of modifiable targets to inform ecologically valid and culturally responsive interventions that will better support Hispanic and Latinx ADRD caregivers.

To our knowledge, this is the first study to provide an in-depth examination of how daily and enduring caregiving-related stress and burden influence the mental health of Hispanic and Latinx ADRD caregivers. This examination is conducted based on the novel application of the protective factor model of resilience. In this model, promotive resources (eg, coping strategies and cultural values) can either reduce risk or improve other protective factors [67,68], and resilience is defined as the capacity for positive adaptation despite adversity [69-71]. Thus, resilience theory provides a strengths-based framework to understand why some caregivers report better mental health outcomes than others despite similar risk exposures. While most studies to date have primarily relied on the stress-process model [72] to inform clinical recommendations, small effect sizes have been repeatedly reported in interventions based on this model [73,74]. The measures included in this study align with domains of the protective-factor model of resilience frameworks including promotive resources/assets and risk factors, that decrease vulnerability to caregiving-related stress and increase resilience over time. Therefore, the results from this study will help inform the development of novel resilience-based interventions to support the well-being of Hispanic and Latinx ADRD caregivers.

In addition, the application of advanced analytical strategies, such as GBTM, allows for a thorough evaluation and understanding of caregivers’ mental health trajectories over time in this understudied population. Further, a subsequent evaluation of the predictive utility of the identified trajectories will allow us to identify which caregivers, under what circumstances, are at risk of developing clinically significant depression and anxiety over time, and which caregivers are resilient. Finally, findings from the Nuestros Días study will translate into key guidance for targeted and culturally responsive behavioral interventions to mitigate the negative effects of caregiving-related stress on Hispanic and Latinx ADRD caregiver mental health.

### Limitations

Although results from the Nuestros Días study will provide compelling directions for future research and tailoring of clinical interventions for caregivers’ mental health, several limitations should be taken into consideration.

First, concerns may be raised regarding the potential burden of completing daily surveys. Although we acknowledge that the study design may appear intensive, it provides a rich in-depth examination of day-to-day changes to the variables of interest, and previous studies [15] using this methodology report good compliance along with unsolicited positive feedback from participants. The daily surveys currently take an average of 10-15 minutes to complete, and the study is purposefully designed to minimize participants’ burdens while supporting the successful completion of the study aims. However, we acknowledge that there could be a potential tendency for participants to provide random answers to DD assessments due to inconvenience. To identify these scenarios, research team members will monitor the DD data to preliminarily identify randomly completed questionnaires.

Second, in the case of low variability in daily mental health measures, we will explore alternative analytical approaches as appropriate (eg, using the single-item questions instead of converting them to a count of symptoms). It is possible to observe low variability in caregivers’ depression and anxiety scores due to either cultural perceptions of mental health or consistently elevated rates of these symptoms at the baseline assessment. To address this concern, we will create a symptom burden score for each period based on the number of days that each participant reported having anxiety and depression symptoms.

Third, the possibility of missing data is a ubiquitous limitation, particularly in longitudinal quantitative research; however, we will use analytical methods [75] (ie, GLMM and GBTM) robust for missing data and will appropriately evaluate patterns of missingness to prevent model misspecification. We also use maximum likelihood estimation to estimate the parameters of the models. Maximum likelihood estimation accounts for data missing at random by estimating the marginal probability of observing a variable given the available data [76]. Finally, we will examine nonparticipant issues (eg, technical failures) and compare the means of observed variables for responders and nonresponders across days, periods, and participants.

Fourth, given the longitudinal nature of the study design, attrition is an additional limitation to consider. To address this concern, we have implemented strategies to keep participants engaged between sampling periods. For example, we mail birthday and holiday cards to participants enrolled in the study. We also send out reminder letters 1 month prior to the 6-month and 12-month data collection waves and place reminder phone calls 1 week before data collection begins. If any mail is returned as undeliverable, we will follow up with an alternate contact provided by the participant upon enrollment. Similarly, after 1 week of 3 unsuccessful attempts to reach the participant at the 6- and 12-month timepoints, we will follow up with the alternate contact. Previous research [77,78] has found that such strategies increase retention over single strategies. If these strategies are unsuccessful, we will identify participants lost to attrition, reopen recruitment, and match the new participants to the lost participants on several demographic variables. Finally, we will use Leigh et al’s approach [79] of specifying an instrumental variable to test whether the likelihood of attrition influences the relationship between key independent measures and mental health outcomes.

### Comparison With Prior Work

Previous studies on Hispanic and Latinx ADRD caregivers have identified important factors that influence the risk of poor mental health outcomes (eg, physical health, family support, and acculturation) [8,80-83]. However, the dynamic relationships among these individual, contextual, and cultural factors, as well as their effects on caregivers’ mental health progression over time, remain relatively unknown among Hispanic and Latinx caregivers. A recent study [62] examining psychological distress among family caregivers of individuals with malignant brain tumors found a trajectory of decreasing psychological distress over 12 months following diagnosis, suggesting psychological adaptation and increased flexibility over time. Such mental health patterns over time are unknown among Hispanic and Latinx ADRD caregivers, which presents a missed opportunity to determine when caregivers are at most risk for developing depression and anxiety.

In addition, while numerous studies [6-10] have observed elevated levels of depression among Hispanic and Latinx caregivers, findings are not consistent across studies, and these discrepancies may be due to the dynamic nature of mental health outcomes, as well as temporal variations in risk or protective factors, yet unexamined in Hispanic and Latinx ADRD caregivers. Further, previous research [61,84-87] has found a high degree of variation in the effects of cultural factors on mental health outcomes. These inconsistencies may be due to the heterogeneity within the culturally, genetically, and regionally diverse Hispanic and Latinx subgroups (eg, Mexican, Puerto Rican, Cuban, and Central American), thus emphasizing the importance of implementing innovative design and analytical strategies to disentangle the within-group variability in this population.

The Nuestros Días study overcomes these limitations and addresses these gaps through the multi-time scale design described and the combination of GLMM and GBTM strategies. Results from this study will identify modifiable intervention targets and at-risk groups, thereby providing foundational knowledge to inform evidence based on the care of Hispanic and Latinx ADRD caregivers.

### Conclusions

In summary, the Nuestros Días study aims to address gaps in the knowledge about the influence of contextual, individual-level, and cultural factors on daily and long-term patterns of mental health of Hispanic and Latinx ADRD caregivers through the implementation of an innovative design and analytical approach. Results from this study will contribute to the development and testing of culturally responsive, resilience-based behavioral interventions to support the well-being of Hispanic and Latinx ADRD caregivers.

## Abbreviations

ADRD: Alzheimer disease and related dementia
DD: daily diary
GBTM: group-based trajectory modeling
GLMM: generalized linear mixed modeling
REDCap: Research Electronic Data Capture

## Data Availability

The data sets generated or analyzed during this study are available from the corresponding author on reasonable request.
